# Adaptive algorithms for shaping behavior

**DOI:** 10.1371/journal.pcbi.1013454

**Published:** 2025-09-12

**Authors:** William L. Tong, Venkatesh N. Murthy, Gautam Reddy

**Affiliations:** 1 School of Engineering and Applied Sciences, Harvard University, Cambridge, Massachusetts, United States of America; 2 The Kempner Institute for the Study of Natural and Artificial Intelligence, Allston, Massachusetts, United States of America; 3 Department of Molecular and Cellular Biology, Harvard University, Cambridge, Massachusetts, United States of America; 4 Center for Brain Science, Harvard University, Cambridge, Massachusetts, United States of America; 5 Joseph Henry Laboratories of Physics, Princeton University, Princeton, New Jersey, United States of America; 6 Physics and Informatics Laboratories, NTT Research, Inc., Sunnyvale, California, United States of America; University of California Santa Barbara, UNITED STATES OF AMERICA

## Abstract

Dogs and laboratory mice are commonly trained to perform complex tasks by guiding them through a curriculum of simpler tasks (‘shaping’). What are the principles behind effective shaping strategies? Here, we propose a teacher-student framework for shaping behavior, where an autonomous teacher agent decides its student’s task based on the student’s transcript of successes and failures on previously assigned tasks. Using algorithms for Monte Carlo planning under uncertainty, we show that near-optimal shaping algorithms achieve a careful balance between reinforcement and extinction. Near-optimal algorithms track learning rate to adaptively alternate between simpler and harder tasks. Based on this intuition, we derive an adaptive shaping heuristic with minimal parameters, which we show is near-optimal on a sequence learning task and robustly trains deep reinforcement learning agents on navigation tasks that involve sparse, delayed rewards. Extensions to continuous curricula are explored. Our work provides a starting point towards a general computational framework for shaping behavior that applies to both animals and artificial agents.

## Author summary

Animals are commonly trained by ‘shaping’ their behavior using a sequence of simpler tasks towards a complex behavior. Many schools of thought have proposed heuristics for shaping based on qualitative principles of reinforcement learning. We introduce a general computational framework for shaping behavior, paying special attention to the constraints faced when training animals. Using machine learning algorithms for planning under uncertainty, we explain why simple strategies fail, provide a normative foundation for existing heuristics, and propose new adaptive algorithms for designing curricula.

## 1. Introduction

Animal trainers “shape” an animal’s behavior towards a complex sequence of actions [1–4]. Shaping is essential when an animal rarely executes the correct sequence during innate behavior. This simple intuition highlights a fundamental constraint that arises due to the curse of dimensionality: learning a complex behavior through random, unguided exploration is impractical when the dimensionality of behavior is large, regardless of which learning rule the animal employs. Shaping tackles this issue by iteratively approximating longer bits of the behavior, limiting the search space at every stage of training.

A shaping protocol typically involves hand-designing a series of simpler tasks leading to the full task during training. The animal is rewarded for successfully completing an assigned sub-task, and the curriculum progresses once the animal is sufficiently good at completing this sub-task [5–9]. Researchers often rely on simple heuristics and prior experience to design these protocols, and it remains unclear whether these heuristics are close to optimal even in simple scenarios, or when these strategies might fail. Such hand curated shaping becomes rate-limiting with increasingly complex tasks and those that need large numbers of subjects. While there have been attempts to automate behavioral shaping [10,11], there has been relatively little effort to systematically optimize the curriculum in learning tasks in the laboratory. Understanding the principles that drive effective shaping, coupled with closed-loop training strategies, would considerably reduce the training time for both laboratory animals and artificial agents, while providing insight into factors that contribute to slow or fast learning [12,13]. Our goal is to develop a general computational framework for shaping behavior that applies to both animals and artificial agents.

In machine learning, the importance of shaping-inspired approaches for training agents was recognized early on [14–20]. More recently, numerous *automatic* curriculum learning (ACL) techniques have been developed for training deep reinforcement learning (RL) agents (reviewed in [21]). Within the ACL framework, an autonomous teacher agent determines the distribution of the student’s tasks based on the student’s past behavior. However, these approaches rely on arbitrary control over the agent’s states [22–24], exploration [25–32] or the reward structure [33–35]. For example, a well-known strategy called potential-based reward shaping [33] modifies the reward function to expedite learning while preserving the optimal policy. Such a procedure is infeasible in experimental situations where the animal has to interrupt its behavior in order to acquire reward. In other cases, these methods assume that the agent’s performance can be measured on a range of arbitrary test tasks [36] or require access to expert demonstrations [24,37,38].

Although these assumptions are reasonable for training artificial RL agents and have demonstrated success in numerous tasks, they are not suitable for training animals. When training animals, we typically have 1) limited flexibility in controlling rewards and exploration statistics, 2) partial observability, as animals can often be evaluated based only on whether they have succeeded or failed on the task (their true “state” remains unknown), and 3) no delineation between training and test trials. In addition, animals often have an innate repertoire of responses and behaviors they may resort to by default, and a training procedure which recognizes and takes advantage of this feature may be more successful.

## 2. Framework

To address these issues, we propose an ACL framework, which we term *outcome-based curriculum learning* (OCL). In OCL, a teacher agent decides the student’s next task based solely on the student’s outcomes, i.e., its history of successes or failures on past tasks, with the long-term goal of minimizing the time to reach a desired level of performance on the final task. By observing and delivering rewards based on binary outcomes, teacher algorithms are task-agnostic, and are applicable for training both artificial agents and animals. Closest to our framework is the teacher-student curriculum learning framework proposed by Matiisen et al. [39,40]. Inspired by the concept of learning progress [41] in developmental psychology, Matiisen et al. [39] propose heuristic strategies where the teacher selects the task on which the student shows the greatest improvement on scores. However, we find below that these heuristics perform poorly compared to our simpler alternatives.

**Fig 1:**
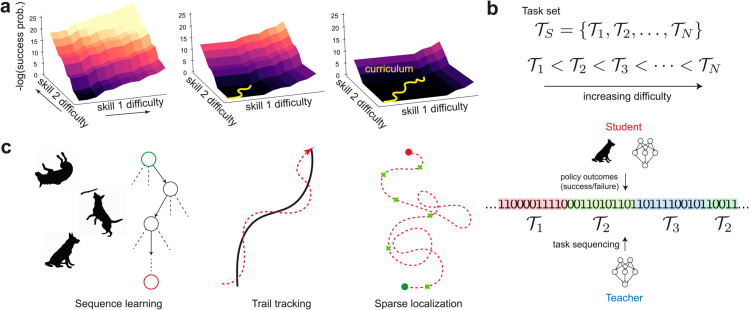
A: Teaching using our OCL framework can be visualized using a difficulty landscape (here, parameterized by two skill axes), which quantifies the student’s success probability for each difficulty level. A student assigned an extremely difficult task will not learn, since they are unlikely to succeed and thus receive little reinforcement. The teacher’s purpose is to adaptively assign tasks (shown in yellow) based on the student’s progress and the difficulty landscape. A successful curriculum charts a short path through the landscape, lowering the difficulty while promoting quick learning. B: Tasks from a pre-defined set are ordered based on their difficulty, as measured by the success probability of a naive agent. An autonomous teacher decides the student’s task (${𝒯}_{1},{𝒯}_{2},{𝒯}_{3}\ldots$) based on the student’s transcript of successes and failures (represented here as 0s and 1s respectively) on previously assigned tasks. C: We apply our OCL framework to three biologically relevant goal-oriented tasks involving delayed rewards: a generic sequence learning task, an odor-guided trail tracking task and a plume-tracking task involving localization to a target based on sparse cues.

To gain intuition, it is helpful to visualize teaching with OCL as ‘navigation’ through an (unknown) difficulty landscape that is shaped by the student’s innate biases towards performing behaviors pertinent to the task. Such a difficulty landscape is illustrated in Fig 1A for a task whose difficulty increases along two independent axes. We define difficulty as the negative log probability of success on a task (here parameterized by the two skill axes) *given* the student’s current policy. The difficulty landscape thus depends on the task as well as the student’s innate biases and learned behavior.

In this manuscript, we consider tasks that can be decomposed into a single difficulty scale. Such tasks lend themselves naturally to a curriculum. A student begins with the simplest version of the task and progresses through difficulty levels (as set by the curriculum) until they succeed at the entire task. In the discrete version of OCL, the experimenter designs tasks and rates them based on their difficulty in discrete levels from 1 to *N* (Fig 1B). A desired threshold level of performance is specified for the *N*th task (the full task). Given this input, the teacher algorithms that we consider below choose the appropriate difficulty level for the student based on their past transcripts. At the start of every interaction, the teacher receives as input a transcript and proposes the difficulty level *k*. The student attempts the task for *T* (fixed) rounds, adding to the performance transcript. This two-way interaction continues until the student attains a satisfactory level of success on the final task.

We first investigate in detail a sequence learning task, where an RL-based student is required to learn the correct sequence of *N* actions (Fig 1C). The sequence learning task encompasses a large variety of behavioral tasks, including tricks such as the roll → fetch → sit sequence described above, numerous Skinnerian tasks, as well as common laboratory behavioral experiments which have a self-initiated trial structure. The difficulty landscape of such tasks is determined by the complexity of the sequence (*N*) and the innate probability that the student will execute the correct action at each step of the sequence. Since the probability of success decreases exponentially with *N*, the agent is unlikely to learn the full task without shaping when *N* is sufficiently large.

The simplicity of the task structure allows us to examine normative teacher strategies using modified Monte Carlo planning algorithms for decision-making under uncertainty. Using insights from these normative strategies, we use differential evolution to design near-optimal heuristics that are agnostic to the task, learning rule, and learning parameters. Next, we apply our method on two novel, naturalistic, sequential decision-making tasks that involve delayed rewards: odor-guided trail tracking and plume-based odor localization (Fig 1C). We show that deep reinforcement learning agents can be trained using our adaptive teacher algorithms to solve these tasks using only a single reward delivered at the end of the task. Finally, we extend this framework to continuous parameterizations of the task, where the teacher has the option of breaking down the task into simpler components.

## 3. Results

### 3.1. Sequence learning

**Fig 2:**
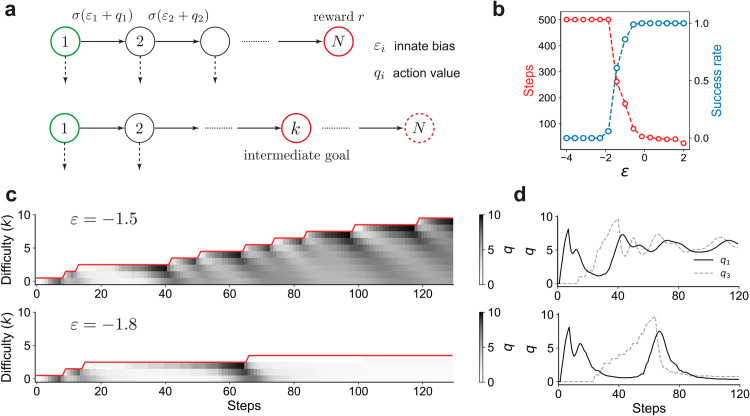
A: The sequence learning setup. In the full task, the student is required to take a sequence of *N* correct actions to get reward. In intermediate levels of the task, the reward is delivered if the student takes k≤N correct actions. $\varepsilon_{i}$ is the innate bias of the student to take the correct action at the *i*th step, prior to training. We assume $\varepsilon_{i}=\varepsilon$ for all *i* unless otherwise specified. B: The incremental teacher (INC) fails once ε≲−1.7. C: The *q* values (in grayscale) for the correct action at each step shown for ε=−1.5 (top) and ε=−1.8 (bottom). The red line shows the assigned task level. Note the striped dynamics in the top row caused due to alternating reinforcement and extinction. In the bottom row, *ε* is too small, forcing learning to stall. D: Time series of *q* values for actions at the first (solid black) and third (dashed gray) steps for the two examples shown in panel C.

In the sequence learning task, a student RL agent begins each trial at a fixed start state and receives a reward *r* if they perform the correct sequence of *N* actions (Fig 2A, see Appendix A in S1 Text for full details). If the student fails to take the correct action at any step in the sequence, the student receives no reward and the episode terminates. The probability that the student takes the correct action at step *i* is given by $\sigma (q_{i}\;+\;\varepsilon_{i})$, where $\varepsilon_{i}$ is the (fixed) innate bias that determines the probability the student will take the correct action before any learning occurs. *σ* is the logistic function. For example, if the agent prior to learning takes *K* possible actions at step *i* with equal probability and only one of them is correct, we have $\varepsilon_{i}=-\log(K\;-\;1)$. The action value *q*_*i*_ is initially set to zero and updated using a standard temporal-difference (TD) learning rule with learning rate *α*.

The sequence learning task naturally splits into discrete difficulty levels: the teacher modulates difficulty by increasing, decreasing or maintaining the step *k* at which the student is rewarded. The innate biases $\varepsilon_{i}$’s play a key role in the dynamics since they determine the probability of success (and thus the rate of reinforcement) when the difficulty level is increased. We assume for simplicity that all $\varepsilon_{i}$’s are equal to *ε*; the general case is considered later. We seek OCL algorithms that minimize the time the student takes to succeed at a rate greater than a threshold *τ* on the full task without prior knowledge of the student’s innate biases and learning parameters.

### 3.2. An incremental teacher strategy is not robust

An intuitive baseline strategy when designing a curriculum is an incremental (INC) approach: the teacher increments the difficulty by one when the student’s estimated success rate $\hat{s}$ exceeds *τ* at the current level. Note that since the success rate changes due to learning, a reasonable estimator $\hat{s}$ should consider recent transcripts yet a sufficient number of them to minimize sampling noise. We consider different estimation procedures for computing $\hat{s}$ and find that an exponential moving average estimator is computationally inexpensive and achieves comparable performance as other more sophisticated methods (Appendix E and Fig B in S1 Text).

INC is stable for large *ε* (Fig 2B). However, INC abruptly and consistently fails when *ε* is below a threshold (ε≲−1.7 in Fig 2B). This value of *ε* corresponds to a naive success rate of ∼15% at each difficulty level. Our results imply that the sparsity of positive feedback when the success rate is below this value to eventually generate a catastrophic failure. Examining the dynamics of the *q* values provides insight into why this catastrophic failure occurs.

Let us first examine *q* value dynamics when the student is required to directly solve the case *k* = 5, where *ε* is chosen such that the student is capable of learning without a curriculum. The dynamics of *q* values exhibit a ‘reinforcement wave’, where actions are sequentially reinforced backwards from the final state to the start [42]. This backward propagation is a generic feature of RL, since the goal acts as the sole source of reward and reinforcement propagates through learning rules that act locally. Now, suppose the difficulty is incremented by one (*k* = 6). Immediately after this change, the student executes the correct sequence of actions until the fifth step, but will likely fail to receive reward as the final step has not been reinforced. These (possibly brief) series of failures produce a long-lasting extinction wave that propagates backwards to earlier steps with dynamics that parallel those of the reinforcement wave. In short, transient failures after every difficulty increment have long-term effects on learning dynamics and success rate.

When visualized over the course of a curriculum, *q* values assume characteristic “striped”-dynamics that emerge due to alternating waves of extinction and reinforcement (top panel in Fig 2C). These striped dynamics reflect the transient failures and eventual successes that follow an increment to higher difficulty when *ε* is larger than the failure threshold. Extinction dominates reinforcement when *ε* is below a critical value, leading to catastrophic unlearning of previous actions and subsequent lack of learning progress. In this event, the agent may eventually learn after a very large number of trials, but extinction negates the benefit of a curriculum by forcing the agent to learn from scratch.

More broadly, reinforcement and extinction effects are related to the value that an agent associates with future actions. In animal behavior, one common and historically salient analogy for these values is motivation. An animal that does well and receives consistent reward is intuitively more motivated than an animal that does not receive reward. Our framework predicts that when a curriculum increases the task difficulty, an animal receives less reward than expected, resulting in a transient dip in motivation. However, when the new task is especially hard, the motivation loss may not be transient, resulting in behavioral extinction. Since extinction is unavoidable after significant increases in difficulty ($e^{\varepsilon }\ll 1$), optimal strategies that are robust in this regime will have to ameliorate this effect while completing the curriculum as quickly as possible. That is, effective curriculum design strategies should achieve an optimal balance between extinction and reinforcement.

### 3.3. Near-optimal teacher algorithms alternate between difficulty levels

To gain insight into near-optimal strategies, we formulate the teacher’s task for the sequence learning task as optimal decision-making under uncertainty using the framework of Partially Observable Markov Decision Processes (POMDPs) [43–45]. Specifically, the teacher decides whether to increase, decrease or keep the same difficulty level based on the student’s past history, and receives a unit reward when the student crosses the threshold success rate on the full task. A discount factor incentivizes the teacher to minimize the time to reach this goal. As when training animals, one challenge is that the student’s true learning state (encoded by the *q* values) are hidden as the teacher receives only a finite transcript of successes and failures on previously assigned tasks. Another challenge is that the teacher is not *a priori* aware of the student’s innate biases and learning rate. Moreover, the long horizon and sparse reward makes planning computationally prohibitive.

**Fig 3:**
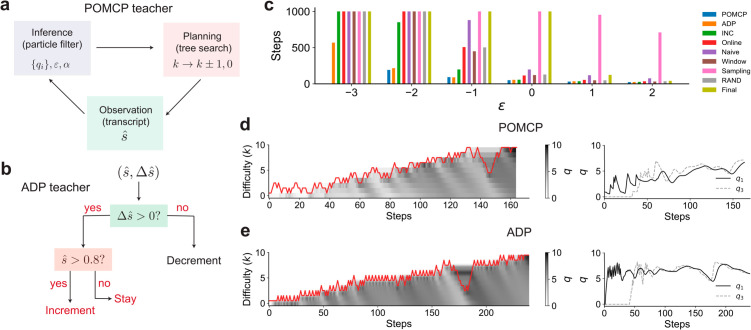
A: An overview of the POMCP teacher, which cycles between inferring the student’s *q* values, innate bias and learning rate based on the transcript and planning using a Monte Carlo tree search. B: The adaptive heuristic (ADP), which employs a simple decision rule to stay, increment or decrement the current difficulty based on the estimated success rate $\hat{s}$ (computed using an exponential moving average over past transcripts). C: POMCP and ADP are comparable and significantly outperform other algorithms [39] when the task is non-trivial (low *ε*), including when INC fails (ε≲−1.7). Here *N* = 10. Note that planning using POMCP is intractable when ε=−3. Barplot means are estimated from 10 repeats. D,E: POMCP and ADP adaptively alternate between difficulty levels, thereby preventing catastrophic extinction. Note the drop in difficulty levels after significant extinction in both cases. Here ε=−2.

To solve this task, we employ an online POMDP solver (called POMCP [46]) that relies on Monte Carlo planning and inference (Fig 3A). This solver plans based on the inferred joint distribution of *q*’s, *ε* and *α*, which is represented as a collection of particles with different parameter values. A planning algorithm based on Monte Carlo Tree Search (MCTS) [47] balances exploration and exploitation to decide the next action. The student’s transcript on the following round is then used to update the joint distribution using Bayes’ rule implemented as a particle filter, after which this cycle is repeated. With sufficient sampling of particles and planning paths, the solver provides a near-optimal adaptive teacher algorithm for the sequence learning task. Due to the large size of our POMDP, the implementation of POMCP is nontrivial, with details in Appendix E and F.

The POMCP teacher exhibits a non-monotonic curriculum, repeatedly reverting back to easier tasks before ramping up the difficulty. The *q* values for earlier steps in the sequence are relatively stable and lack the alternating reinforcement and extinction dynamics that we observe for the INC teacher (Fig 3D). This robustness extends to *ε* values lower than the critical value at which INC fails (ε=−2 in Fig 3C). Indeed, as shown in the example in Fig 3D, the POMCP teacher recognizes and compensates for significant extinction by rapidly decreasing the difficulty, increasing difficulty only after sufficient re-learning occurs.

### 3.4. A heuristic adaptive algorithm achieves near-optimal curriculum design

The POMCP teacher’s strategy suggests simple principles to overcome extinction while making learning progress. Specifically, a robust teacher algorithm has to 1) increase difficulty when the estimated success rate ($\hat{s}$) is sufficiently large (similar to INC), 2) continue at the same difficulty level when the success rate is below this threshold value as long as the student continues to learn ($\Delta \hat{s}>\mu$), and 3) decrease difficulty if the student begins to show signs of significant extinction ($\Delta \hat{s}<\mu$) for some *μ*. These three principles motivate our choice of a decision-tree-based teacher algorithm that uses $\hat{s}$ and $\Delta \hat{s}$ as features. The precise splits and leaves of the trees can be optimized using various search procedures. More complex trees can be constructed by taking into account second or higher-order differences of the success rate. For the sequence learning task, we find that the features $\left(\hat{s}(t),\Delta \hat{s}(t)\right)$ are adequate to produce a successful teacher, which we term Adaptive (ADP). We optimize the decision tree using differential evolution (Fig 3B, see Appendix B in S1 Text for details). Note that this optimized ADP is used for all benchmarks below with no additional tuning.

The ADP teacher shows dynamics similar to POMCP, mitigating extinction waves by alternating between difficulty levels (Fig 3E). We benchmark ADP against INC, POMCP and four algorithms proposed by Matiisen et al. [39] (Fig 3C). These latter four algorithms are based on the principle of maximizing *learning progress* [41]: a student should attempt the difficulty level at which they make the fastest progress (as measured by the slope of the learning curve on a particular task). The algorithms differ in how progress is measured and how tasks are sampled based on their relative progress.

ADP is competitive with POMCP (for the range of parameter values that POMCP can be feasibly evaluated) and significantly outperforms the other algorithms for small values of *ε*, which is the regime where curriculum design is non-trivial and baseline algorithms such as INC fail. Moreover, ADP is robust when the innate biases are not equal (Fig A in S1 Text). Since our OCL framework is task-agnostic and model-agnostic, ADP can be directly applied to other tasks and artificial agents provided that sub-tasks are arranged on a discrete, monotonic difficulty scale.

Our ADP teacher algorithm is similar to ‘staircase’ procedures that effectively extend INC with a lower threshold, and decrement the difficulty level when this low threshold is reached [48,49]. A key distinction in our approach is that decrements are based on the change in success rate rather than a second threshold on success rate. While implementing a low threshold on $\hat{s}$ is within the expressive capacity of our ADP framework, we find that our evolved teacher instead relies on $\Delta \hat{s}$ in its decision making (Fig 3B).

### 3.5. Performance of ADP on deep RL tasks with delayed rewards

To examine whether ADP can design curricula for complex behaviorally relevant tasks and learning models, we train deep RL agents to solve two navigation tasks with delayed rewards: odor-guided trail tracking and plume-source localization.

Dogs are routinely trained to track odor trails, and various heuristics have been developed by trainers to efficiently teach dogs [50]. In a successful trail tracking episode, the student begins with a random orientation from one end of the trail and receives a reward only when they get to the other end of the trail. Trails are long, meandering and broken so that the agent is highly unlikely to get to the end through random exploration and should thus learn a non-trivial strategy to actively follow the trail and receive reward.

**Fig 4:**
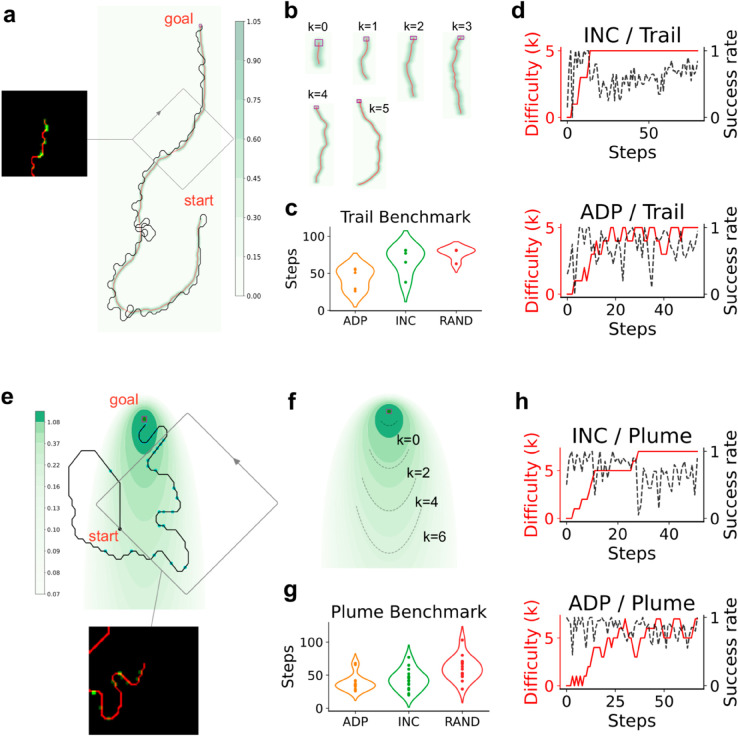
Deep reinforcement learning agents trained using a curriculum solve navigation tasks with delayed rewards. A: The trail tracking paradigm. A sample trajectory of a trained agent navigating a randomly sampled odor trail. The colors show odor concentration. The inset shows the egocentric visuospatial input received by the network, where the agent’s location is in red and odor detections are in green. B: Sample trails from the six difficulty levels. C: ADP outperforms INC and RAND (each teacher-student interaction is a step). The agent does not learn the task without a curriculum. Results are plotted from 5 repeats. D: The success rate of the agent in finding the target over training (black dashed line) for INC and ADP. The curriculum is shown in red. Note the significant forgetting shown by the student trained using INC approach compared to ADP. E-G: As in panels A-D for a localization task. The agent is required to navigate towards a source which emits Poisson-distributed cues whose detection probability decreases with distance from the source (colored in green on a log scale). Results are plotted from 15 repeats.

The trail tracking paradigm (Fig 4A–4D) provides a natural split of tasks onto a difficulty scale. We design a parametric generative model for trails where the parameters control the length, average curvature and brokenness of the trails (Appendix C in S1 Text). Samples of trails along tasks of increasing difficulty are shown in Fig 4B. We develop a deep RL framework for trail tracking, where the tracking student uses its sensorimotor history of sensed odor and self-motion to modulate their orientation in the subsequent step (see Appendix C in S1 Text for full details). Sensorimotor history is encoded using a visuospatial, egocentric representation (Fig 4A), so that the student has a memory determined by the size of the visuospatial observation window. The student uses a convolutional neural network architecture which is trained using Proximal Policy Optimization (PPO) [51].

We observe the following outcomes. Without a curriculum, a student trained on the final level of the task never attains reward within the training cutoff (about 1000 curriculum steps). ADP outperforms both INC and a curriculum (RAND) that randomly chooses from the task set (Fig 4C). Their curricula show that ADP alternates as in the sequence learning task, presumably mitigating extinction effects associated with the transition to more difficult tasks. INC is comparable to ADP but experiences a greater degree of forgetting as seen in the longer time it spends at the highest difficulty level (Fig 4D). The path of an agent tracking the trail is shown in Fig 4A. The agent exhibits a preference for localizing at the edge of the gradient. When it encounters a break, the student performs repeated loops of increasing radius until it re-establishes contact with the trail. A detailed analysis of the student’s tracking behavior during trail tracking is postponed to future work.

Next, we extended this framework to a localization task (Fig 4E–4G) inspired by naturalistic plume tracking [52,53] and sound localization tasks. In each episode, the student begins at a random location a certain distance from a target whose (fixed) location is unknown. A unit reward is delivered when the student localizes at the target. The student receives sparse, Poisson-distributed cues from the target with probability that depends on the relative location to the target. These cues provide information about the location of the target, which can be used by the student to solve the task (see Appendix C in S1 Text for full details). The delayed reward and sparse cues provide a challenge for training agents without a shaping protocol. We consider a curriculum where the difficulty scale is determined by the rate of detecting a cue at the student’s initial position, as well as the student’s distance from the target (Fig 4F). Similar to the trail tracking setting, results recapitulate the better performance of ADP compared to INC and RAND (Fig 4G, 4H).

### 3.6. Continuous curricula

Our analysis up to this point assumes discrete curricula. A consequence of discrete curricula is that an unexpectedly large jump in difficulty from one level to the next can stall learning. In such situations, an animal trainer has the option of decomposing the task further and proceed with an INC approach. However, if the jump from one level to the next is too small, the student will progress in small steps while the teacher incurs a temporal cost on unnecessary evaluations. On a continuous curriculum, an optimal teacher has to adjust difficulty increments such that they reflect the student’s innate biases. Here, we explore preliminary ideas for designing continuous curricula using a continuous extension of the sequence learning task and a concomitant modification of the student’s learning algorithm (see Appendix D in S1 Text for more details).

**Fig 5:**
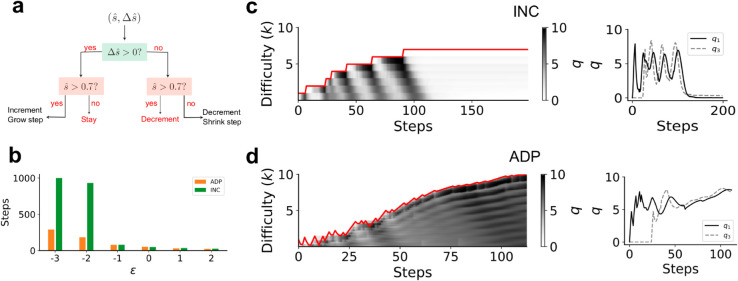
Algorithms for designing continuous curricula A: Decision tree showing the continuous version of ADP which includes actions that “grow” and “shrink” the increments between continuously parameterized difficulty levels. See the text for more details of the task in the continuous setting. B: ADP significantly outperforms INC when the task is difficult (low *ε*). Barplot means are estimated from 10 repeats. C,D: The *q* values plotted as in Fig 3D, 3E. Similar to the discrete setting, INC shows catastrophic extinction and never learns the task for sufficiently small *ε*. Continuous ADP first decreases increment size and smoothly increases the difficulty level while balancing reinforcement and extinction.

We consider a continuous ADP teacher modified to accommodate the particulars of a continuous curriculum. At the start of an interaction, the experimenter proposes an initial “rough guess” for the difficulty increment used by the teacher. As the ADP teacher progresses, it tweaks the size of this increment based on the student’s performance. In addition to the three actions (increase, decrease and maintain difficulty), we introduce a second set of three actions: increase, decrease and retain the increment interval (the teacher selects from nine actions at each step). As in the discrete case, we use differential evolution to find the best decision tree (Fig 5A). Fig 5C, 5D shows trajectories for the continuous ADP teacher, which compares favorably with INC in benchmarks (Fig 5B).

## 4. Discussion

From Skinner’s missile guidance pigeons [54] to laboratory rodent experiments to state of the art artificial RL agents, curriculum design plays a foundational role in training agents to solve complex tasks. Here, inspired by behavioral shaping, we propose adaptive algorithms that can be applied for training both animals and artificial RL agents, and evaluate our algorithms on RL agents trained to solve biologically-inspired tasks. In a sequence learning task, dual waves of reinforcement and extinction modulate the student’s performance, necessitating a careful shaping strategy that balances reinforcement and extinction. A naïve teacher, INC, fails to prevent extinction when students encounter large jumps in difficulty. A near-optimal teacher strategy (POMCP), discovered by formulating teaching as optimal planning under uncertainty, relies on frequent alternations between the current and previous task difficulty levels, which ameliorates extinction. Inspired by this observation, we use differential evolution to design a decision-tree-based heuristic algorithm, ADP. ADP is much more efficient and achieves performance comparable to that of POMCP, significantly outperforms other algorithms on the sequence learning task and requires no fine-tuning for the task or student. ADP outperforms other curriculum strategies when applied to train deep RL agents on complex, naturalistic navigational tasks.

We focus primarily on cases where the curriculum can be decomposed into rigid, discrete difficulty levels. Real-world tasks can often be further broken down when student’s encounter a bottleneck. We explore one continuous generalization of ADP that relies on finite approximations to continuous intervals, coupled with a *K*-step TD learning rule. The continuous setting poses a distinct challenge: since the teacher *a priori* does not know whether the student can solve an incrementally harder version of the task, estimating this through a transcript takes additional samples and thus incurs a temporal cost. Infintesimal increases in difficulty are not optimal. On the other hand, large jumps in difficulty will stall learning. We expect competitive algorithms to appropriately balance these two factors; a more exhaustive exploration of continuous OCL algorithms will be considered in future work.

For many real-world tasks, there are multiple axes that must all be optimized simultaneously. For example, a tennis player has to learn and compose multiple elements – footwork, racquet motions, tactics – in order to improve general playing skill. In the trail tracking setting, we have simplified all such factors (length, average curvature, brokenness of the trails) into a single difficulty scale, when ideally, the teacher should choose how to modulate the difficulty along each factor. In such a setting, reinforcement and extinction may take place along each axis of the task. A naive generalization of our framework would simply apply a teacher algorithm to each task in parallel, though correlations between tasks may either help or hinder the learning process. Generalizing our teacher algorithms to settings where there are multiple relevant skills represents an exciting future direction.

There are numerous examples of laboratory tasks where the framework can be applied, often using methods similar to clicker training to provide immediate feedback and to break down a complex task into simpler sub-tasks. For example, in an olfactory discrimination task involving odor mixtures, mice were trained to detect a target odor in the presence of up to fourteen other background odors [8]. In that work, the authors used a hand-designed curriculum to train mice starting from easier tasks (single odors), and gradually move to tasks where the number of components in the mixture increases. The complexity of the mixture provides a natural linear scale for difficulty. The decision to move up the curriculum to the next level was dependent on the experimenter’s observations and decisions. To apply our framework to this task, task difficulty would first be defined in experiments as the average number of components in the background mixture. The animal’s performance is gauged by measuring the success rate over a fixed set of trials of a certain difficulty, adding to an existing ‘transcript’. The algorithms in our framework take as input the transcript and produce an output that specifies whether the task difficulty (here, the mixture complexity) should be increased, decreased or kept the same.

A second example setting is the virtual reality navigation task [55,56], where head-fixed mice are trained to navigate in a virtual corridor by manipulating the ball that they stand on. Mice run down the virtual corridor, paying attention to different number of visual cues on the left and right walls. Training for this task is done by gradually increasing the length of the corridor (see Supplementary Fig 1 in [56]). Training is reported to take several weeks or even months, and requires frequently lowering the difficulty to the previous stage if animals are confused and do not perform well in an incremented task level. Such decisions of moving the curriculum are again made by experimenters.

In a third example, Poddar et al. [11] introduced an automated procedure to train rats on different tasks including making center-out joystick movements and making precisely timed lever presses. Training occurred in their home cages using a fully automated training system, whose parameters appear to be experimenter determined. Rats took several weeks and tens of thousands of trials to learn these tasks (see Figs 2 and 4 in [11]). Applying our framework to both these examples would require translating aspects of the task (such as the length of the corridor in the former case or the timing precision necessary to receive reward in the latter case) to discrete difficulty levels. Measuring a set of binary outcomes for a particular difficulty level adds to the transcript, which is then read by the teacher algorithm to set subsequent difficulty levels.

Such laboratory tasks often involve extensive training lasting for weeks or more [9]. Concepts like task difficulty levels, innate bias, behavioral reinforcement, and extinction have natural analogs when using shaping to train animals. It is often unclear whether lengthy training is due to the innate difficulty in animals learning the tasks at hand, or inefficient curriculum design [13]. Developing better teacher algorithms for animal training may result in significant savings in time and cost to produce well-trained subjects. Importantly, using a common protocol across laboratories may also reduce variability in behavioral and physiological outcomes of trained animals. We anticipate future work that applies our teacher algorithms to a laboratory animal training, measuring how well our principles hold in an applied setting.

In addition to practical benefits for laboratory research, any demonstration of more rapid training of animals will also shed light on their capabilities and limits of learning. Gradual shaping we discuss here may also be related to gradual introduction of more intuitive coincidences that exploit an animal’s priors to allow more rapid learning [13]. Such hand-crafted shaping is common in laboratory experiments [5–9], but more precise quantitative descriptions of behavioral learning algorithms such as ours opens the possibility of designing near-optimal teaching strategies in more general scenarios, similar to the POMCP formulation that we have developed here for a RL-based student. Optimistically, such formulations might even impact curriculum design for human students.

## Supporting information

S1 Text Appendices with additional experiments and details. (PDF)
