# Supplementary material for: Adaptive algorithms for shaping behavior
Source: PLoS Comput Biol. 2025 Sep 12;21(9):e1013454. doi: 10.1371/journal.pcbi.1013454 (PMC12448964; doi:10.1371/journal.pcbi.1013454)
Supplement: S1 Text — (PDF) [file pcbi.1013454.s001.pdf]

Appendix A: Sequence learning

We describe in detail the specifics of the sequence learning task, and our model of a student. The task consists of a series of discrete choices, inspired by the setup in [1]. At each timestep, the student must select the correct action to advance. Selecting the wrong action at any timestep terminates the episode without reward. If the student advances N times in a row, where N is the pre-determined length of the environment, the episode terminates with fixed reward R.

Selecting the correct action is determined by the student's Q-values, which are updated using a simple TD learning rule described below (Section A 1). Though the model is constructed with binary actions (correct / incorrect), it describes any situation where actions can be partitioned into correct and incorrect groups. The probability of selecting a correct action is modified using an additional parameter ε, which we describe further below. In this way, the task models any setting where a student must execute a consecutive sequence of correct actions, capturing a large portion of tasks classically suitable for curriculum learning.

See Fig 2A for a graphical depiction of the task. An MDP that summarizes this task can be given as

- **State space**: integers $1, 2, \dots N$
- **Action space**: move forward, halt
- **Transitions**: For states s, s' and action a, we have the following transition probabilities:

$$\begin{aligned}
&\Pr(s' = i+1 \,|\, s = i, a = \text{move forward}) = 1, \text{for } i < N \\
&\Pr(s' = \varnothing \,|\, s = N, a = \text{move forward}) = 1 \\
&\Pr(s' = \varnothing \,|\, s, a = \text{halt}) = 1, \text{for any } s
\end{aligned}$$

- **Reward**: the reward function is simply $R(s = N, a = \text{move forward}) = R$ and 0 otherwise. For all sequence learning tasks, reward is fixed at $R = 10$. Note, for sufficiently large R, the specific choice of R will not qualitatively change the learning dynamics of the student. The student will learn quicker, but the learning curves will have the same shape. If R is chosen too small, the student experiences a bottleneck and its Q-values never saturate.

This setup lends itself naturally to a curriculum, where larger values of N correspond with more difficult tasks. The goal of the teacher is to propose a number of intermediate tasks indexed by their lengths $n_1, n_2, n_3, \dots$ such that the student succeeds at the final task of length N.

Note, we assume here that the task has already been broken down into N discrete steps. For a generalization to the continuous setting where N can vary along the real numbers, see Appendix D.

1. Model of the student

A student in the sequence learning setting is modeled as an Expected SARSA RL agent [2, 3]. For each state i, the student has two Q-values: q_i^{forward} and q_i^{halt}, which correspond to the correct and incorrect actions, respectively. Because the incorrect halt action is never reinforced, we always have that $q_i^{\text{halt}} = 0$. For simplicity, we omit specifying q_i^{halt} explicitly, and refer to q_i^{forward} as simply q_i.

In a standard softmax policy, the probability of moving forward from state i is given by

$$\pi(\text{forward} \,|\, i) = \sigma(q_i) \tag{1}$$

where σ is the sigmoid function, $\sigma(x) = \frac{1}{1+e^{-x}}$.

In the real world, different students have different propensities for learning a task. One lab mouse may be easier to train than another. One dog may have a better sense of smell, and track a trail more efficiently than another. One student may learn quicker than another, and follow a correspondingly accelerated curriculum. Modeling a student's propensity for a task is therefore an essential consideration when designing curricula.

In our sequence learning setting, we incorporate this property by introducing a bias term ε. This term serves two related purposes:

1. **Model the student's innate propensity for success**: every student has a different set of innate talents and preferences. Modulating ε allows us to simulate students with abilities that are more or less aligned with the task.
2. **Model the student's extrinsic chance of success**: for more complex tasks, a student often has to choose one correct option among many incorrect choices. Modulating ε controls the student's initial probability of success, capturing extrinsic factors that may aid or hinder the student's progress.

To model these effects, we adjust the student's policy to be

$$\pi(\text{forward} \,|\, i) = \sigma(q_i + \varepsilon_i), \tag{2}$$

where q_i is updated through learning, but ε_i remains fixed. The value of ε_i can be different for different steps i, but in our experiments we typically set ε_i to be the same across all i and simply write it as ε.

The value of q_i is updated using the Expected SARSA update rule. Upon taking the correct action from state i, the value of q_i becomes

$$q_i \leftarrow q_i + \alpha(r + \gamma\langle q_{i+1}\rangle_\pi - q_i) \tag{3}$$

Note, the expectation $\langle q_{i+1}\rangle_\pi$ is computed including bias ε, so

$$\langle q_{i+1}\rangle_\pi = \sigma(q_{i+1} + \varepsilon)q_{i+1} \tag{4}$$

To simplify the analysis, we assume the student has an infinite horizon, i.e., the discount factor $\gamma = 1$.

Appendix B: Teacher algorithms

We describe in greater detail our three primary teacher algorithms: Incremental (INC), POMCP, and Adaptive (ADP). Specific hyperparameter settings for all algorithms are attached at the end of this supplement.

1. Incremental teacher

An intuitive strategy for building a curriculum is to assume an incremental approach: the teacher proposes tasks of incrementally increasing difficulty as the student masters each successive level. For the sequence learning task, if the goal is to learn a task of length N, the teacher would start with a task of length 1. Once the student masters length 1, the teacher proposes length 2. Once the student masters length 2, the teacher proposes length 3. And so on until the student masters length N. See Algorithm 1 for a concrete description of this process. See Appendix E for details on how student's are evaluated.

Algorithm 1 Incremental teacher for discrete curriculum

```
Require: target difficulty N, threshold success rate τ
  n ← 1
  while n ≤ N do
      s_n ← EVALUATE(Student)
      if s_n > τ then
          n ← n + 1
      else
          Train Student on task level n
      end if
  end while
```

2. POMCP teacher

Just as the student is a reinforcement learning agent, the teacher can also be considered a reinforcement learning agent attempting to discover an optimal policy for a partially-observable Markov decision process (POMDP) [4]. Informally, the teacher's observation space consists of the student's outcome history up to the current time. After observing the student perform for a time, the teacher must take an action: namely selecting the next level of task to present. The process is partially observable because the teacher lacks full knowledge of the student's state, and must infer the student's true level of performance from the outcome history. Discovering an optimal policy for the resulting POMDP would shed insight on how an optimal curriculum should look for any particular student.

Formally, we define the following POMDP underlying the teacher's decision process:

- **State space**: states consist of a 3-tuple (q, ε, α) where q represents all of the student's learnable Q-values, ε represents the student's innate ability bias, and α represents the student's learning rate. In effect, the state is the set of values that fully define the student's ability to perform the sequence learning task.
- **Action space**: integers $1, 2, \dots N$, which correspond to the next task level that the student will see. To ensure that solving the POMDP remains tractable, we limit the action space to 3 values: 1) increment the task difficulty, 2) decrement the task difficulty, 3) keep the same task difficulty
- **Transitions**: transitions from (q, ε, α) to $(q_{\text{new}}, \varepsilon, \alpha)$ can be sampled given a transcript by applying the SARSA update rule in Equation 3. For POMCP, we do not need an explicit probability distribution across transitions, only the ability to sample from it.
- **Reward function**: for a pre-determined success threshold τ, if a student's rate of success at the final task exceeds τ, the teacher receives a fixed unit reward R. Concretely, the student's rate of success at a sequence of length k is

$$\mu_k = \prod_{i=1}^{k} \sigma(q_i + \varepsilon) \tag{1}$$

  Let $s^* = (q^*, \varepsilon, \alpha)$ be a 3-tuple for which $\mu_N > \tau$. Then any transition that terminates in s^* receives reward R, and the episode terminates. No other transition is rewarded, or terminates the episode (unless a pre-determined max number of iterations is exceeded).
- **Observation space**: the teacher observes the student's transcript $\mathbf{h} = (h_t, h_{t-1}, \dots, h_{t-T+1})$ since the last interaction, where $h_i = 1$ if the student succeeded, and 0 otherwise. Because the transcript prior to the last interaction is encoded in the estimated parameters (q, ε, α), we do not need the entire history back to $t = 0$, and can keep just the last T episodes.

We use the Partially Observable Monte-Carlo Planning (POMCP) algorithm [5] to approximately solve this POMDP. POMCP operates by using a particle filtering algorithm to estimate the student's underlying learning state (Appendix E). This estimate is paired with a Monte Carlo tree search procedure to select approximately optimal curriculum levels for the student to learn next. The resulting procedure identifies approximately optimal actions for a POMDP. Please see Silver and Veness [5] for a detailed overview of the POMCP algorithm. Our specific hyperparamter settings are enumerated in Appendix F.

The resulting teacher proposes curricula that differ from INC in several striking ways (Fig 3). Most importantly, rather than monotonically increment the curriculum by steady intervals, the POMCP teacher backtracks

to earlier levels in an oscillatory movement. By alternating between two or more levels, the POMCP teacher pre-empts an extinction wave before it propagates all the way back to the start, erasing the student's progress. In this way, the teacher encourages new reinforcement waves to form at higher levels while mitigating the impact of extinction, and successfully trains a student even for severely low ε.

3. Adaptive teacher

A key insight from the success of the POMCP teacher is that "backtracking" is essential to the success of a curriculum. Rather than increment in gradual, monotonic steps as the student learns, it is important to interleave easier levels periodically so as to counter extinction effects. Whereas POMCP decides the points at which to backtrack through a blackbox search procedure, the Adaptive teacher explicitly optimizes for points to backtrack.

ADP works by learning a decision tree that decides whether to increment or decrement the current task level. As input, the teacher receives a history of success rates $s^{(1)}, s^{(2)}, \ldots s^{(t)}$ estimated from the student at each step up to the current time t. A *first-order* Adaptive teacher will additionally compute $\Delta s^{(1)}, \Delta s^{(2)}, \ldots \Delta s^{(t)}$ where $\Delta s^{(i)} = s^{(i)} - s^{(i-1)}$ (and $s^{(j)} = 0$ for $j < 1$). When making a decision, the Adaptive teacher then compares the current success measures $(s^{(t)}, \Delta s^{(t)})$ against a decision tree whose leaf nodes correspond to one of three possible actions: 1) increment task level, 2) decrement task level, and 3) stay at current task level. See Fig 3 for an example of what this decision tree looks like.

The precise splits and leaves of the trees can be optimized using any number of popular search procedures [6–9]. The Adaptive teacher can be further customized with additional "features." For example, we could also implement a *second-order* teacher, which includes features $\Delta^2 s^{(i)} = \Delta s^{(i)} - \Delta s^{(i-1)}$, or arbitrary features $\Phi(s^{(i)}) = f(s^{(1)}, s^{(2)}, \ldots s^{(i)})$ for some arbitrary function f. For this simple sequence-learning task, we find that the features $(s^{(t)}, \Delta s^{(t)})$ are adequate to produce a successful teacher.

Optimizing the Adaptive teacher proceeds as a coordinated ascent. The procedure begins with an initial, reasonable set of actions selected by the experimenter. Differential evolution [6] is used to evolve the precise splits in the tree, followed by a comprehensive search through the entire space of possible actions. These two steps, evolution followed by action search, alternate until converging on a final tree.

Appendix C: Odor tracking

During olfactory navigation, an animal encounters a series of discrete odor measurements in pursuit of a target. Odors can be terrestrial or airborne, that is, concentrated along a single thin trail or diffused along turbulent air currents, respectively. Examples include a dog tracking the scent of deer, a moth following pheromones to find a mate, or a mouse pursuing fragrant hints of last night's leftovers. This behavior is prevalent and essential across species, necessary for navigation, foraging, and mating [10–12].

Below, we detail two related deep reinforcement learning tasks that simulate odor-guided navigation: 1) surface-borne odor trail tracking and 2) airborne odor plume tracking. For both settings, we employ the same deep RL framework to recreate naturalistic tracking environments.

1. RL framework

Given the successes of various deep RL algorithms on video game tasks [13], we encode sensory-motor history using a visuospatial pixel representation. This representation allows the agent to learn both a sensory stack for interpreting the image inputs as well as a navigational policy for finding the target.

a. Observation space

For both trail and plume tracking, the agent is centered on a flat, 2D surface without landmarks or obstructions. Odor is either distributed along a thin trail (for trail tracking) or diffused in simulated plume (for plume tracking). As the agent navigates this landscape, it encodes its position history together with the last few odor detections within each individual image observation.

The observation space consists of a 50×50 grid of pixels and 3 color channels per pixel, which produces a full RGB image. Although the agent's position is recorded continuously during each episode, when producing the pixel observations, it is discretized to the pixel grid. The three color channels of the image encode different information. The first channel (i.e. the "red" channel) encodes the agent's position history. The second channel (i.e. the "green" channel) encodes the agent's odor measurements. The last channel (i.e. the "blue" channel) is left unused.

At each timestep, the agent moves to a different location and performs an odor measurement. Position history is recorded as a thin continuous line in the image observation's red channel, linearly interpolating between discontinuous points. Odor history remains discrete, and is depicted as colored patches at each location where the agent performed an odor measurement. This difference reflects the fact that motion through a space is continuous, but odor measurements occur only when the agent "sniffs" the environment. Hence, odor measurements in history must be discrete. The strength of the odor is proportional to the pixel value in the green channel. The

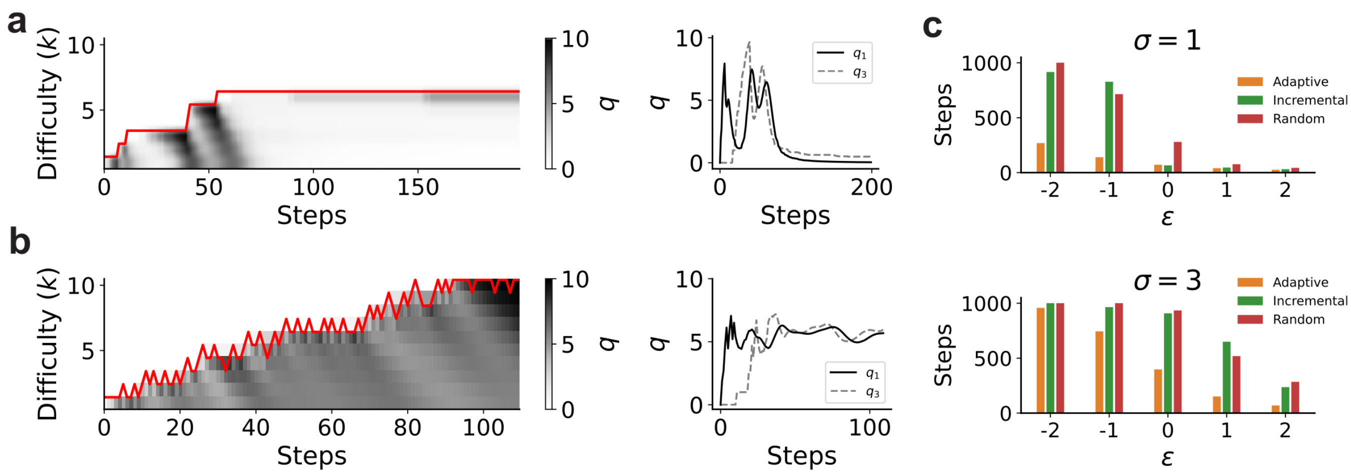

FIG. A: (a,b) q value dynamics for INC (panel a) and ADP (panel b) when ε_i values for each difficulty level are heterogeneous. Specifically, $\varepsilon_i = \varepsilon + \sigma\eta$, where η is a standard normal random variable. These examples are run with $\varepsilon = -1$ and $\sigma = 1$. (c) Performance comparison between ADP, INC and a random strategy for $\sigma = 1$ and $\sigma = 3$. Barplot means are estimated from 10 repeats.

image overall remains centered on the agent, that is, it is egocentric with respect to the agent's position and heading. As the agent moves in a particular direction, the whole "viewport" moves and rotates with the agent, leaving a track that spreads out away from the center recording the agent's past. See Fig 4A for an example observation that the agent receives.

2. Action space

At each timestep, the agent can take one of three actions: 1) move left, 2) move right, 3) move forward. Choosing the move left (or right) action will shift the agent's heading by 45° in the corresponding direction. After any heading updates, the agent's position is then incremented by three units along its new direction.

3. Reward

If the agent lands within 3 units of the target (either the end of a trail or source of a plume), the episode terminates with a large, fixed reward. Otherwise, if the agent fails to reach the target within a predetermined maximum number of iterations, the episode terminates without reward. No other actions are reinforced, nor are any negative rewards ever applied.

Note, the reward scheme is intentionally sparse. If an agent were to attempt a long trail without additional aid, it will fail to converge towards a successful strategy due to the lack of sufficient reinforcement signals. A traditional approach to addressing this sparsity is through reward shaping [14, 15], a process whereby the designer supplies supplementary rewards that guides the agent towards successful behavior. However, reward shaping is difficult to implement in practice, may require significant assumptions about the student, and different shaping strategies may have unpredictable impacts on the agent's overall behavior [2, 16]. Instead, we use curriculum learning to overcome the sparsity issue. By starting the agent on short, easy trails for which the reward scheme is not sparse, then gradually lengthening the distance to the target, the agent naturally learns a successful tracking strategy without the heavy-handed tuning required for reward shaping.

4. Model

We use Proximal Policy Optimization (PPO) [17, 18], a popular deep RL algorithm that achieves state of the art on a wide variety of discrete and continuous tasks. The agent uses a deep convolutional neural network to extract operable features from each image observation, followed by several fully-connected layers to infer state values and output actions. For specific implementation details and hyperparameter settings, please see Appendix F.

5. Trail tracking

Surface-bound odor trails are long, thin segments of concentrated odor with minimal diffusion through the air. To sense these trails, an animal must be relatively close, and may not always sustain contact. Further, these trails often have breaks: significant stretches during which odor is absent, and the animal must execute a search strategy to regain contact. Terrestrial trail-tracking is therefore a highly nontrivial behavior.

To construct naturalistic trail geometries, we use the procedure described in Reddy *et al.* [19]. Trail characteristics are modulated with the following parameters:

- **Length**: the distance between the agent's starting position and the target.
- **Width**: the scale at which odor diminishes along the axis perpendicular to the trail. With width parameter σ^2 and a distance x from the trail, the concentration of odor o is proportional to

$$o \propto \exp\left(-\frac{x^2}{\sigma^2}\right)$$

- **Heading**: the angle between the agent's initial heading, and the direction of the target.
- **Shape**: the shape of the trail is governed by two parameters: curvature and diffusion rate. Curvature governs how the high-level shape of the trail evolves over time. Diffusion rate governs how "kinky" the trail is on short intervals.
- **Breaks**: a trail can have one or more breaks of variable length, along which there is no odor.

The agent perceives the exact magnitude of odor at its current location, which is represented in the magnitude of the green color channel in each image observation. This is in contrast to Reddy *et al.* [19], which uses a Poisson-based odor detection model. See Fig 4A for a plot of an example trail, including the trajectory of a successful agent.

For the purpose of building curricula, the difficulty of a trail is rated by a combination of its length, width, and breaks. The heading of the trail is allowed to vary across the entire compass rose, and the shape parameters are held constant across all episodes. Curricula are discretized by hand, with predetermined difficulty levels that specify particular parameter combinations. The specific difficulty levels are described in Appendix F. Because our teachers rely on discrete curricula, this extra manual intervention is necessary to apply these algorithms. In an ideal case, teacher algorithms should generalize to continuous curricula, which we explore further in Appendix D. At the start of every episode, a new trail is sampled using the parameter combination specified by the curriculum, and the agent must track an unseen trail from the beginning.

6. Plume-source localization

Often times, odor diffuses through the air in turbulent plumes. For humans, this mode of olfaction is perhaps more commonly experienced than terrestrial odor trails: the smell of baked goods at the local pastry shop, fragrance from a spring garden, or the comforting scent of your home are all plume-based odors. Like terrestrial trails, odor plumes are essential for survival and reproduction across animal species. However, tracking the source of an odor plume presents its own unique difficulties. Plumes tend to be rarefied and clumpy, where air turbulence partitions regions of odor concentration into random, disconnected patches. An animal attempting to locate the source of a plume must infer its location based on sporadic contacts and indirect cues.

To simulate naturalistic plumes, we use the model described in Vergassola *et al.* [20]. Odor plumes are shaped through the following parameters:

- **Wind speed**: a high wind speed produces long, elongated plumes. A low wind speed produces squat, round plumes. Zero wind produces a spherical plume.
- **Start rate**: the initial rate of detections at the agent's starting position. A low start rate implies that the agent will start further away from the source of the plume, and have a correspondingly harder task.
- **Particle properties**: additional parameters like diffusivity, lifetime, and emission rate are related to the properties of individual particles, and influence the overall shape of the plume.

In contrast to the terrestrial trails, where the agent deterministically perceives the exact magnitude of odor at its current location, the odor detection model in the plume setting is probabilistic and discrete, accounting for the random influence of turbulence. Odor detection is Poisson-distributed, with the rate given by the plume model. See Fig 4E for an example plume, including the trajectory of a successful agent.

For the purpose of building curricula, the difficulty of a plume is rated by its start rate, with lower start rates corresponding to more difficult trails. To ensure the agent is always downwind of the source, the agent's location is initialized within a fixed sector of a particular start rate. As in the terrestrial trail setting, because our teacher algorithms can only accommodate discrete curricula, we discretize the start rate into discrete levels for curriculum learning, with the specific levels given in Appendix F. A framework for handling continuous curricula is explored further in Appendix D. The agent's starting *location* is re-sampled at the start of every new episode, though the starting *rate* remains the same for a particular task level. Particle properties and wind speed remain fixed for the entire duration of training.

Appendix D: Continuous curricula

Introducing continuous curricula is a complex and nuanced topic. In this Appendix, we introduce a set of preliminary ideas for addressing the issue, but defer a more complete study to a future paper.

1. Continuous sequence learning

It is not immediately obvious how to generalize the sequence learning task to a continuous curriculum. Here, we propose one approximate scheme that preserves many (but not all) of the intuitions from discrete sequence learning, though it is by no means the only possible scheme.

We begin with the following notion. In the discrete sequence setting, for an untrained student with fixed bias ε, the probability that the student advances from step n to step $n+1$ is given by $\pi(n+1|n) = \sigma(\varepsilon)$. Suppose the student is allowed to move continuously. Then the probability that the student first moves to $n + \frac{1}{2}$, then from $n + \frac{1}{2}$ to $n + 1$ must also be $\sigma(\varepsilon)$ overall. Hence, we must maintain that $\pi(n+1/2|n) \cdot \pi(n+1|n+1/2) = \sigma(\varepsilon)$. In other words, there exists a value $\varepsilon_{1/2}$ such that $\pi(n+1/2|n) \cdot \pi(n+1|n+1/2) = \sigma(\varepsilon_{1/2})\sigma(\varepsilon_{1/2}) = \sigma(\varepsilon)$. In this example, we can see quite clearly that

$$\varepsilon_{1/2} = \sigma^{-1}\left(\sqrt{\sigma(\varepsilon)}\right)$$

In the general case, if a student with *effective* bias ε moves an interval $\Delta x \ll 1$, then we can identify a value $\varepsilon_{\Delta x}$ such that $\pi(n + \Delta x|n) = \sigma(\varepsilon_{\Delta x})$, where

$$\varepsilon_{\Delta x} = \sigma^{-1}\left(\sigma(\varepsilon)^{\frac{1}{\Delta x}}\right) \tag{1}$$

If we allow $\Delta x \to 0$, then we approach a continuous curriculum. In the results that follow, we set $\Delta x = 0.01$.

In this way, we transfer much of the same intuition from the discrete sequence learning task. For an *effective* task length N and bias ϵ, which matches N and ε from a discrete curriculum, the student operates on a "continuous" environment with $N_{\Delta x} = \frac{N}{\Delta x}$ and $\varepsilon_{\Delta x}$. Ideally, a student operating on $(N_{\Delta x}, \varepsilon_{\Delta x})$ should perform identically to a student operating on (N, ε), as long as each curriculum increment is $1/\Delta x$. However, if we apply Equation 3 directly to update the student, we run into an important issue: only the previous Q-value is updated. For the continuous student, each Q-value represents an infinitesimal slice of ability. Applying the old rules as-is means we update only singular, infinitesimal slices at a time on the student. Intuitively, improving at a particular level should improve multiple Q-values simultaneously, and in a fashion that remains consistent with the discrete case. We model this effect by making a small change to the student's update rule.

2. K-step student

For a student on our continuous approximation of the simple sequence learning task, the number of Q-values grows as the unit of discretization Δx decreases. Indeed, for a curriculum of effective length N, the number of Q-values becomes $N_{\Delta x} = \frac{N}{\Delta x}$. Without changes to the update rule described in Equation 3, the student is limited to updating one Q-value at a time. As $\Delta x \to 0$, the number of Q-values to-be-updated approaches infinity, requiring infinite time for any reinforcement wave to propagate to the start.

Intuitively, as a student develops one particular Q-value, neighboring Q-values should also be updated. After all, because $N \approx N - \Delta x$, if q_N changes, $q_{N-\Delta x}$ should also change by approximately the same amount. Note, progress may be symmetric such that $q_{N+\Delta x}$ also change by a similar amount. Such an approach implies some form of a smoothing strategy for the update rule. However, for simplicity and consistency with the discrete case, we assume asymmetric updates where only Q-values less than the current task level N have an opportunity to be updated. A natural way to incorporate this intuition is to change from a single-step update rule as described previously to K-step expected SARSA.[2] That is, rather than update each Q-value using its immediate next neighbor, the update rule now becomes

$$q_i \leftarrow q_i + \alpha\left(\sum_{j=1}^{K} \gamma^{j-1} r_j + \gamma^K \langle q_{i+K} - q_i \rangle\right)$$

where r_j is the reward observed j steps ahead of the current step i. Because reward is only dispensed at the very end of a successful run, we have that $r_j = 0$ for $j < N_{\Delta x}$. Using a discount $\gamma = 1$ as before, the update simplifies to

$$q_i \leftarrow q_i + \alpha(r_K + \langle q_{i+K} - q_i \rangle_\pi) \tag{2}$$

In effect, rather than updating from the immediate next-neighbor Q-value, the K-step student now updates using the Q-value K steps ahead. Hence, upon encountering a reward, all previous K Q-values are updated rather than just the immediately preceding one. On a later pass, as the student approaches this block of updated Q-values, the preceding block of K values also receives updates as the intervals overlap. If we allow $K = 1/\Delta x$, the range of updated values correspond precisely with the updated values in the discrete sequence learning task.

For sufficiently high ε, the continuous K-step student corresponds precisely in learning speed with the discrete student. However, one important difference is the propagation of mistakes. In the discrete case, if the student halts, the error is not backpropagated. Rather, the (unwritten) Q-value associated with choosing the halt action is "updated," and remains 0. However, in the K-step scenario, because all previous K Q-values are updated, if the student halts, the last $K-1$ Q-values are depressed slightly with a zero update, initiating a secondary extinction wave distinct from the ones generated through curriculum changes. Hence, learning is slower and more difficult for continuous students, and the potential for extinction waves to erase all progress correspondingly higher. The situation is exacerbated by low ε, in which

the probability of halting is higher. An easy way to fix this situation is to simply alter the learning rules such that halting actions do not propagate depressed Q-values. However, it remains unclear whether this effect is a feature or bug — perhaps it is more realistic for repeated failures to depress the performance of the student, rather than leave performance unimpacted. Perhaps the correct action is to modify the discrete student such that errors also propagate. Ultimately, we leave this distinction as an additional layer of complexity for the continuous student, and an additional obstacle an optimal teacher must overcome.

3. Teacher algorithms

We explore generalizations of both the Incremental and Adaptive teachers. Unfortunately, POMCP does not scale to the massive size of the POMDP in the continuous case. We rely on the behavior of the Adaptive teacher to give a sense for what optimality looks like in this setting. Note, none of the algorithms from Matiisen *et al.* [21] generalize at all to continuous curricula, so we do not investigate them further in this setting.

Incremental generalizes directly to this setting. We allow the curriculum to increment in fixed intervals of $1/\Delta x$, monotonically increasing the task difficulty as the student progressively attains mastery. The procedure otherwise remains identical to the one described in Algorithm 1. Fig 5 shows trajectories for the Incremental teacher adapted for the continuous setting.

a. Continuous Adaptive teacher

The Adaptive teacher is similar in spirit to its discrete counterpart but extended to accommodate the particulars of a continuous curriculum. At the start of an interaction, the experimenter proposes an initial "rough guess" at an appropriate increment interval for the teacher to use. Such an increment can be considered a prior that the experimenter assumes about the difficulty of a task. For the continuous sequence task, we use an initial increment of $1/\Delta x$. As the Adaptive teacher progresses, it tweaks the size of the increment according to the student's performance. Hence, rather than just three actions (increment, decrement, stay), we introduce a second set of three actions: increase the increment interval, decrease the increment interval, retain the same interval. Adjustments to the interval are made multiplicatively by a predetermined percentile.

With this adjustment, at every iteration, the Adaptive teacher must now select from one of nine possible actions: an `increment`, `decrement`, `stay`, paired with a `grow interval`, `shrink interval`, and `keep interval`. To select the correct action, one can use the same optimization procedure described in Section B 3 to learn the best actions for each situation. Fig 5 shows trajectories for the Adaptive teacher adapted for the continuous setting (compare to Fig 3E).

b. Benchmarks

See Fig 5 for a comparison of Incremental and Adaptive teachers on the continuous sequence learning task. Adaptive has a decisive edge over Incremental, particularly for high N and low ε. Random and final-task-only curricula are not plotted, as learning fails to occur even for the easiest tasks pictured.

Overall, intuitions from the discrete case translate naturally to the continuous case, though learning overall tends to be more difficult in the latter. We presented here only an initial exploration of one particular continuous generalization. Future work will need to examine more deeply nuances particular to the continuous case, what optimal teachers may look like in this setting, and validate these results on a naturalistic task like trail tracking.

Appendix E: Estimation methods

A central aspect of outcome-based curriculum learning is that the teacher does not have access to the student's internal parameters. Rather, the teacher must estimate intrinsic qualities of the student through extrinsic observables alone. In our context, the critical internal parameter of the student we estimate is its true success rate s_n for a task of difficulty n. For example, in the case of the Incremental teacher, if s_n exceeds some threshold τ, the student advances. Otherwise, the student remains on the current task level. In the case of Adaptive, s_n and Δs_n are used as inputs to a decision tree that decides the student's next task.

In all these cases, the teacher has access only to the student's transcript of successes and failures: $\mathbf{h} = (h_1, h_2, \ldots, h_t)$, where $h_i = 1$ if the student succeeded on round i, and 0 otherwise. Given this transcript, the teacher must construct an estimate $\hat{s}_n(\mathbf{h}) \approx s_n$. In the main text, we consider only a simple exponential moving average (EMA) approach to computing $\hat{s}_n$. In this appendix, we describe the EMA in further detail, and explore two alternative estimation procedures motivated by a Bayesian approach.

1. Overview

Under the student model described in Section A 1, the true success rate of the student on a task of length n is given by

$$s_n = \prod_{i=1}^{n} \sigma(q_i) = \prod_{i=1}^{n} \sigma(q_i + \varepsilon) \tag{1}$$

However, because the underlying parameters q_i and ε are unknown to the teacher, the quantity s_n must be estimated from the student's transcript of successes and failures. Below, we compare three estimation procedures for s_n:

1. **Exponential moving average**: we apply an EMA to the student's transcript to estimate s_n. This estimate functions as a baseline with which to compare our other two, Bayesian motivated approaches

2. **Beta posterior inference**: we assume the successes and failures in a student's transcript are drawn iid from a Bernoulli distribution. Because the student's success rate evolves as it learns, s_n is a nonstationary quantity, and so this assumption cannot hold over long periods of time. However, to simplify posterior inference, we assume that negligible training occurs over short time periods, and that s_n is locally stationary.

3. **Particle filtering**: we relax the local stationarity assumption from Method 2 and develop a particle filtering algorithm to estimate s_n. Particles are sampled from priors over q_i and ε, then forward-simulated to yield posterior samples over s_n.

In the following sections, we develop each method in detail, and compare their ability to estimate s_n. To remove the confounding influence from complex teacher strategies, we use only the Incremental teacher as a framework for comparing these estimation procedures, and later compare Incremental (with sophisticated estimation techniques) to the gold-standard POMCP teacher.

2. Exponential moving average

Recall that at time t, the teacher observes a transcript of the student's performance $\mathbf{h} = (h_1, h_2, \dots h_t)$, where $h_i = 1$ if the student succeeded on episode i, and 0 otherwise. The EMA algorithm constructs an estimate $\hat{s}_n$ through the following recursive update rule as new observations h_t are made:

$$\hat{s}_n \leftarrow \begin{cases} 0 & t = 0 \\ (1-\gamma)h_t + \gamma \hat{s}_n & t > 0 \end{cases} \tag{2}$$

which corresponds to the unrolled equation

$$\hat{s}_n(\mathbf{h}_t) = \sum_{k=0}^{t} \gamma^k (1-\gamma) h_{t-k}$$

The parameter $\gamma \in [0, 1]$ is chosen by the experimenter prior to computing the EMA, and controls the degree to which past observations are discounted.

The exponential moving average is a simple, non-parametric approach to estimating a nonstationary quantity. See Fig B in S1 Text for a plot of EMA estimates compared to the true underlying success rate on a task with length $N = 10$, using the teacher model described above. We compare EMA across different discounts γ and across students with different bias parameters ε. Higher values of γ resulted in smoother estimates, though with a greater lag behind the true value. Lower values of γ resulted in noisier estimates, though with less lag. For $\gamma < 0.5$, the estimates became meaningless as they tended to alternate between extremes. Overall, a discount of $\gamma = 0.8$ seems to be the most appropriate, and tracks the true success rate with reasonable consistency.

3. Beta posterior inference

We next consider a simple Bayesian approach to this estimation problem. One challenge of estimating s_n is that this quantity is nonstationary. As the student learns the task, s_n changes over time. However, to make the analysis simpler, we can make the (big) assumption that over short intervals, s_n is essentially stationary. In this case, suppose s_n is approximately stationary over the time interval $[t-k, t]$, for some small integer k. Then for a transcript $\mathbf{h} = (h_1, h_2, \dots h_t)$, we have

$$h_t, h_{t-1}, \dots h_{t-k} \overset{\text{iid}}{\sim} \text{Bernoulli}(s_n)$$

Hence, if we impose the prior $s_n \sim \text{Beta}(\alpha, \beta)$, the posterior on s_n becomes

$$s_n | \mathbf{h}_{(t-k):t} \sim \text{Beta}\left(\alpha + n_1(\mathbf{h}_{(t-k):t}), \beta + n_0(\mathbf{h}_{(t-k):t})\right)$$

where n_1 and n_0 count the number of ones and zeros respectively. This form suggests an estimator based on the posterior mean $\hat{s}_n = E[s_n | \mathbf{h}_{(t-k):t}] = \frac{\alpha + n_1}{\alpha + \beta + k}$ In this setting, we assume a uniform prior $s_n \sim \text{Beta}(1, 1)$. Selecting the value k is somewhat trickier. We would ideally like to select a k that is as small as possible, so as to ensure s_n does not change too much over the interval $[t-k, t]$. At the same time, if k is too small, our posterior has a higher variance, and the confidence in our estimate is correspondingly lower.

To address the latter issue, let us first consider the lowest value of k we can tolerate. Suppose we use a threshold τ such that if $s_n > \tau$, then the student advances to the next level. Suppose we would like to be confident at the level c such that s_n exceeds τ before allowing the student to advance. Then we stipulate that $p(s_n | \mathbf{h}_{(t-k):t} > \tau) > c$. In the best case scenario, all observations in $\mathbf{h}$ are ones. The minimum k we can tolerate is therefore the smallest k such that $p(s_n | \mathbf{h}_{(t-k):t} > \tau) > c$ is true when $\mathbf{h} = 1, 1, \dots 1$. To compute this k, we observe that

$$p(s_n | \mathbf{h}_{(t-k):t} < \tau) < 1 - c$$

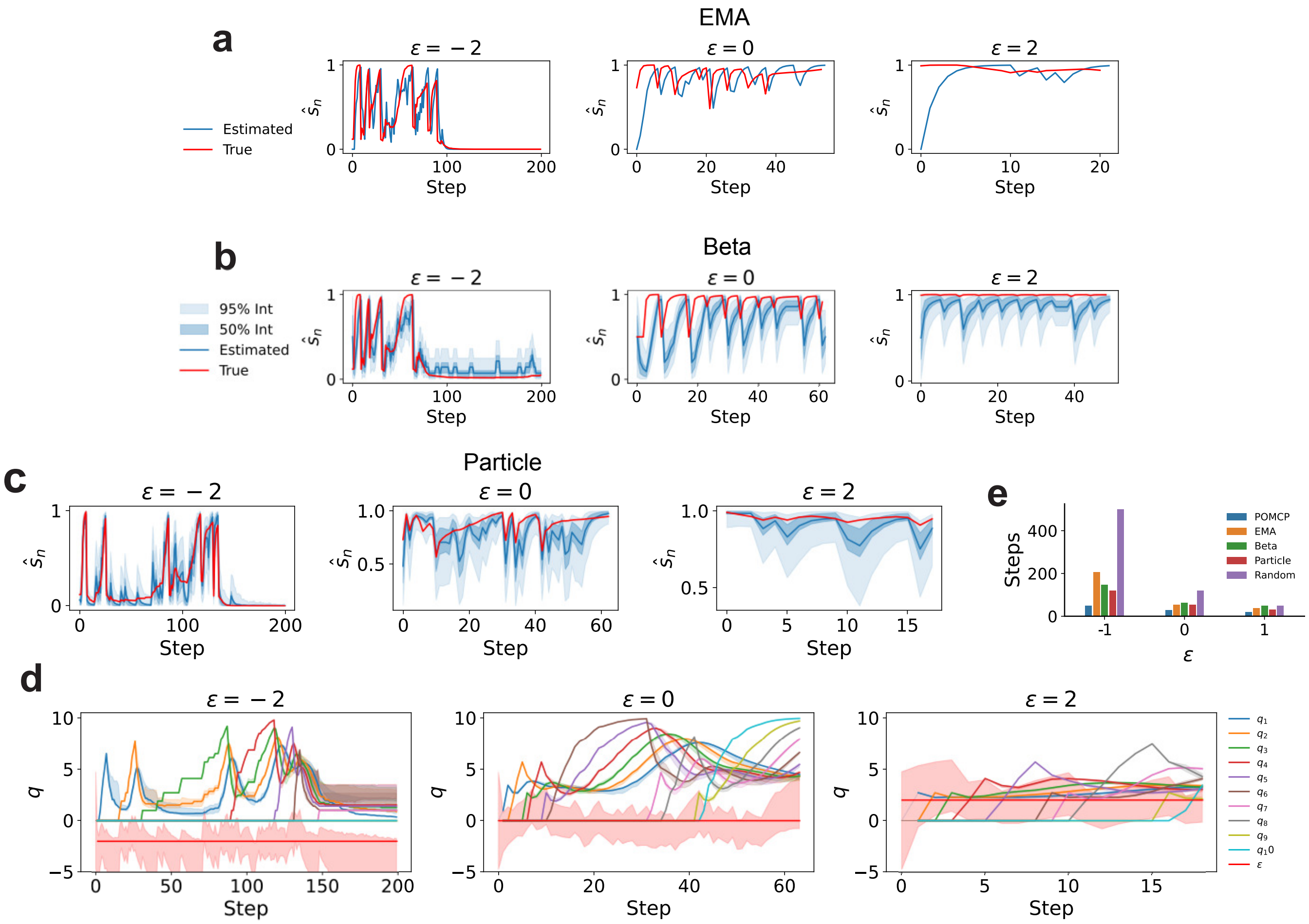

FIG. B: The following plots compare the performance of each estimation method under an INC teacher. (a,b,c) The true and estimated success rate when an exponential moving average (a) beta posterior inference (b) and a particle-filter-based approach (c) is used for estimation. In panels b,c the 50% and 95% confidence intervals are also shown. (d) The q values estimated by POMCP, with 95% intervals. (e) Performance comparison between ADP using the three different estimation methods. We use EMA in the main text due to its simplicity and efficiency. Barplot means are estimated from 10 repeats.

and

$$p(s_n|\mathbf{h}_{(t-k):t} < \tau) = I_\tau(k+1, 1)$$

where $I_\tau(\alpha, \beta)$ is the incomplete Beta function with upper limit τ. From here, we see that $I_\tau(k+1, 1) = \tau^{k+1} < 1 - c$. Solving for k yields the final result

$$k > \frac{\log(1-c)}{\log \tau} - 1$$

so we can establish a lower bound on k as $k_{\text{low}} = \lfloor \frac{\log(1-c)}{\log \tau} - 1 \rfloor$. In practice, we use a confidence $c = 0.5$, which corresponds to when the median of the posterior exceeds the threshold τ, and yields reasonable results. Note, because s_n changes for different difficulty levels n, k should not be so large that it dips into episodes where n was smaller. Hence, after the student advances, we must wait at least k_{low} rounds before evaluating the student. For the EMA algorithm, this was a non-issue because EMA would adapt its estimate of the success rate to the current statistics. In contrast the Beta posterior procedure relies on an underlying stationary quantity, and so would be obviously invalid if it uses samples from s_{n-1} in its estimation of s_n.

To determine what the upper bound on k should be, it is unclear how to select a reasonable upper-bound analytically. However, because s_n is presumably increasing over long timescales, the further back we look, the lower our estimate $\hat{s}_n$ will become. Hence, we establish a coarse upper bound $k_{\text{high}} = 3k_{\text{low}}$, and evaluate $p(s_n|\mathbf{h}_{(t-k):t} > \tau) > c$ for every k in between k_{low} and k_{high}. If at any point this expression evaluates to true, we advance the student to the next level. Because $\hat{s}_n$ decreases for larger values of k, there should ideally be a single k between k_{low} and k_{high} that balances a large enough sample that produces a high-confidence estimate, and a small enough sample that does not yield an underestimate on s_n. As before, k_{high} should not be so large as to contaminate the data with samples from s_{n-1}. The prefactor 3 was chosen as a hyperparameter setting that

seems to work well in practice, though other factors may be used.

See Fig B in S1 Text for a plot of the Beta posterior estimate, along with the true estimates of the student's performance. In general, the Beta posterior estimate tracks the trajectory of the student's true success rate, but underestimates it systematically. This may be in large part because our assumption of local stationarity on the scale of k is invalid. Significant learning likely occurs even within just k_{low} steps, motivating the need to develop an approach that does not rely on this tenuous assumption.

4. Particle filtering

Our final estimation approach does not assume any stationarity in s_n; rather, it estimates s_n by simulating the underlying learning dynamics using a particle filtering algorithm. Specifically, we estimate posteriors on the student's Q-values q_i and innate bias parameter ε, then construct an estimate of s_n based on Equation 1.

The particle filtering algorithm proceeds as follows:

1. **Sample initial particles from the prior**. We apply a uniform prior on ε over the interval $[-5, 5]$, which encompasses the range of reasonable values ε can take. We apply a point-mass prior centered on 0 for all q_i, because each q_i is initialized to 0 for every student. An initial set of particles $(\mathbf{q}_j^{(0)}, \varepsilon_j^{(0)})_{j=1}^M$ are sampled from these priors. In the simulations, we find that $M = 1000$ is sufficient.

2. **Simulate forward dynamics**. For each particle, we simulate T trials of the student using the learning rules described in Equation 3, obtaining a set of updated parameters $(\mathbf{q}_j^{(1)}, \varepsilon_j^{(1)})$ along with a set of corresponding simulated transcripts $(\mathbf{h}_j^{(1)})$

3. **Filter particles consistent with observation.** After receiving an observed transcript $\mathbf{h}$ from the student, we keep all particles j such that $\mathbf{h}_j^{(1)} = \mathbf{h}$. Note, contrary to previous descriptions where we assume $\mathbf{h}$ corresponds to the entire history of the student's performance, here we assume $\mathbf{h}$ corresponds only to the student's trials since the last interaction, and $|\mathbf{h}| = T$. If T is small, checking direct equality works well. However if T is large, the probability that an observation will match a simulated transcript diminishes accordingly (even if the underlying parameters match), in which case we might compare a summary statistic on $\mathbf{h}$ like the mean. In our case, $T = 3$, which is sufficiently small to check for equality directly.

4. **Resample remaining particles.** For our remaining particles, to remove any outliers, we perform a resampling weighted by each particle's likelihood. For a particle $(\mathbf{q}_j^{(1)}, \varepsilon_j^{(1)})$ and observation $\mathbf{h}$, if the student's learning rate α is sufficiently small, the likelihood of the particle can be given as

$$p(\mathbf{h}|\mathbf{q}_j^{(1)}, \varepsilon_j^{(1)}) = \hat{s}_n^{n_1(\mathbf{h})}(1 - \hat{s}_n)^{n_0(\mathbf{h})}$$

where $\hat{s}_n$ is the particle filtering estimate of the true success rate at level n, and follows from equation 1

$$\hat{s}_n(\mathbf{q}, \varepsilon) = \prod_{i=1}^{n} \sigma(q_i + \varepsilon) \tag{3}$$

With these weights, all particles are resampled until we have a set of the original size M particles.

5. **Particle reinvigoration.** Particularly for long runs, ε will tend to drift over time as unexpected observations are encountered (the Q-value parameters tend to remain fairly close, as we will see below). To forestall any posterior collapse, it is essential to reinvigorate ε. We use a simple reinvigoration strategy where a small random jitter is applied to every ε parameter. Specifically, we use a normally-distributed jitter centered at 0 with variance 0.25.

6. **Repeat.** Encountering one observation yields a set of particles $(\mathbf{q}_j^{(1)}, \varepsilon_j^{(1)})$, which represents samples from the posterior $\mathbf{q}, \varepsilon \,|\, \mathbf{h}^{(1)}$. Upon encountering a second observation, we repeat our calculations from step 2 using the current posterior sample to obtain a new set of particles $(\mathbf{q}_j^{(2)}, \varepsilon_j^{(2)})$, which represents samples from the posterior $\mathbf{q}, \varepsilon \,|\, \mathbf{h}^{(2)}, \mathbf{h}^{(1)}$. This process is repeated for all new observations as they arrive.

Using this particle filtering algorithm, we generate posteriors on the parameters q_i and ε. At step i, the posterior samples $(\mathbf{q}_j^{(i)}, \varepsilon_j^{(i)})_{j=1}^M$ can be used to construct posterior samples on estimated success rate $\hat{s}_n$ using Equation 3. From here, we can adopt the same approach as before, and if more than c proportion of the posterior samples are greater than a predetermined threshold τ, we advance the student to the next level. As for the Beta posterior inference approach, we use $c = 0.5$.

See Fig B in S1 Text for a plot of the estimated success rate during runs using the particle filtering algorithm. In general, the estimates fall quite close to the true success rate, outperforming both of the previous methods. Fig B in S1 Text plots 95 percent intervals on the posterior samples of the underlying parameters, compared to the true values for each parameter. The particle filtering approach tends to capture the true values well, confirming that its inference is accurate.

5. Comparison of estimation procedures

Fig B in S1 Text shows a comparison across all teacher algorithms, including the POMCP teacher. The POMCP

teacher represents the approximately optimal teacher, and shows the lower bound that all other teachers approach. In general, our simple incremental teacher model approaches the optimal, though of course does not quite attain it, particularly for lower ε and higher difficulty levels N. Somewhat surprisingly, it also appears that the accuracy of an estimation method does not matter critically, as all three estimation methods produce students with more-or-less the same efficiency, though the particle-filtering method retains a slight edge.

In application, the particle filtering approach is no longer tractable as a success estimation method in more complex settings like our naturalistic tracking tasks. Between the remaining two options (EMA and Beta posterior inference), EMA is faster and simpler, with fewer tunable hyperparameters, while retaining good performance. Hence, we select EMA as our estimation method of choice in the main text.

Appendix F: Implementation details

All code is available on GitHub: `https://github.com/wtong98/automated-curriculum-learning`

1. Sequence learning

In the sequence learning setting, the student is an expected SARSA agent with the following parameters:

Parameter	Value
Reward	10
Learning rate	0.1
Discount	1
Episodes per interaction	3

2. Success estimation

INC and ADP use an exponential moving average (EMA) to measure the success rate of the student, with discount factor $\gamma = 0.8$. (Alternative approaches are discussed in Appendix E.)

In the sequence learning setting, the EMA averages over the student's history of successes and failures. In the deep RL setting, multiple students are run simultaneously to parallelize sample generation during rollout. The transcript across all parallel students are averaged in time to produce a single mean transcript for EMA.

3. Incremental teacher

The Incremental teacher has a single parameter: the success rate τ beyond which the student should advance to the next level. We use $\tau = 0.95$ for the sequence learning task, and $\tau = 0.7$ for the deep RL tasks, selected through a hyperparameter sweep.

4. POMCP teacher

The POMCP teacher uses the following parameter settings:

Parameter	Value
Particles	5000
Discount	0.9 - 0.95
MCTS stop threshold	0.01
MCTS explore factor	1
Reinvigoration probability	1
Reinvigoration scale	0.5

We implement POMCP using the particle filtering algorithm described in Appendix E, with an additional state variable α that represents the student's learning rate. Particle reinvigoration applies only to the estimation of the student's innate bias parameter ε. For a particle estimate p_ϵ, reinvigoration proceeds as $p_\varepsilon \leftarrow p_\varepsilon + \eta$, where $\eta \sim \mathcal{N}(0, \sigma)$. Estimation of the student's learned Q-values tend to be highly accurate, and hence do not require reinvigoration.

A random rollout policy does not scale well to low ε for our problem. Instead, rollouts are computed following an "Incremental policy" – the decisions that an Incremental teacher would make. In other words, if the success rate of the student based on the sampled parameters exceeds $\tau = 0.95$, the increment action is chosen. Otherwise, the stay action is chosen. An Incremental policy proved to work sufficiently well even for low ε tasks, though given the sensitivity of Incremental teachers to very low ε, a more intricate policy may perform more efficiently in this regime.

5. Adaptive teacher

Fig 3 shows the decision tree used by the Adaptive teacher on the sequence learning task (for both homogeneous and heterogeneous ε). The decision tree learned by the Adaptive teacher for the continuous case is shown in Fig 5. For the sequence learning task, optimizing the Adaptive teacher proceeds as a coordinated ascent. The procedure begins with an initial set of actions guessed by the experimenter. Differential evolution [6] is used to evolve the precise splits in the tree, followed by an exhaustive search through the space of possible actions. These two steps, evolution followed by action search, alternate until converging on a final tree.

The decision tree used by ADP on the deep RL tasks was tuned by hand, and proceeds as follows. For an estimated success rate $\hat{s}$ and change in success rate $\Delta\hat{s}$

- If $\hat{s} > 0.7$ and $\Delta\hat{s} \geq 0$: increment

- If $\hat{s} < 0.65$ and $\Delta\hat{s} < 0$: decrement
- Otherwise: stay

6. Matiisen teachers

We use a grid search to identify optimal hyperparameters for the teacher algorithms described in Matiisen *et al.* [21]. The final parameters we used are:

- **Online**: $\alpha = 0.05, \beta = 0.34$
- **Naive**: $\alpha = 0.16, \beta = 3.8$
- **Window**: $\alpha = 0.26, \beta = 4.83, k = 10$
- **Sampling**: $\alpha = 0.1, k = 3$

7. Trail tracking

Trails are generated using generalized worm-like chain ensembles, using the procedure described in Reddy *et al.* [19]. The parameters used to sample each trail are

Parameter	Value
Width	5
Diffusion rate	0.02
Curvature radius	70
Heading	0 - 2π
Break point	0.5 - 0.6

The `break point` parameter specifies the segment of trail over which there is no odor, and is expressed as a proportion of the trail's total length. The schedule used to produce Fig 4 varies based on the trail's length. The specific lengths used are: $\begin{pmatrix}10 & 30 & 50 & 70 & 90 & 100\end{pmatrix}$

The agent is a PPO [17] deep reinforcement learning model with the following hyperparameters

Parameter	Value
Samples per rollout	8192
Batch size	256
Epochs	5
Learning rate	1e-4
Feature model	Nature CNN[22]
Action model	MLP
Value model	MLP
Entropy coefficient	0.1
Discount	0.98
GAE weight	0.9
Gradient clip range	0.2
Max gradient norm	1
Value function clip range	0.36

"MLP" refers to a multi-layer perceptron with 2 layers, 128 units per layer, and ReLU activations.

The agent receives as input a pixel observation of the world with a square view distance of up to 40 units in the horizontal or vertical directions, scaled up by a factor of 2 to produce the direct pixel observations. At each step, the agent advances three units in the forward direction, or 45 degrees to the left or right. The agent's heading rotates left/right by the same angle. The agent is allowed up to 200 steps before the episode terminates.

8. Plume tracking

Plumes are generated using the plume model in Vergassola *et al.* [20]. The parameters used to generate each plume are

Parameter	Value
Length scale	20
Diffusivity	1
Emission rate	1
Particle lifetime	150
Wind speed	5
Sensor size	1

The schedule used to produce Fig 4 varies based on the starting odor detection rate. The specific rates used are computed as $1/r_k$, where $r_k = 0.5 + 0.75k$ and k increases from 0 to 7, for a total of 8 difficulty levels.

As before, the agent is a PPO [17] deep reinforcement learning model, with the following hyperparameters

Parameter	Value
Samples per rollout	8192
Batch size	256
Epochs	5
Learning rate	1e-4
Feature model	Nature CNN[22]
Action model	MLP
Value model	MLP
Entropy coefficient	0.25
Discount	0.98
GAE weight	0.9
Gradient clip range	0.2
Max gradient norm	1
Value function clip range	0.36

"MLP" refers to a multi-layer perceptron with 2 layers, 128 units per layer, and ReLU activations.

The agent's mechanics are identical to those of the trail case. The max steps the agent can take in the environment scales as 3 times the starting distance to the odor source.

[1] G. Reddy, A reinforcement-based mechanism for discontinuous learning, Proceedings of the National Academy of Sciences **119**, e2215352119 (2022).

[2] R. S. Sutton and A. G. Barto, *Reinforcement Learning: an Introduction* (MIT press, 2018).

[3] H. Van Seijen, H. Van Hasselt, S. Whiteson, and M. Wiering, A theoretical and empirical analysis of expected sarsa, in *2009 IEEE Symposium on Adaptive Dynamic Programming and Reinforcement Learning* (IEEE, 2009) pp. 177–184.

[4] K. J. Åström, *Optimal Control of Markov Processes with Incomplete State Information I*, Journal of Mathematical Analysis and Applications, Vol. 10 (Elsevier, 1965) pp. 174–205.

[5] D. Silver and J. Veness, Monte-carlo planning in large pomdps, Advances in Neural Information Processing Systems **23** (2010).

[6] R. Storn and K. Price, Differential evolution-a simple and efficient heuristic for global optimization over continuous spaces, Journal of Global Optimization **11**, 341 (1997).

[7] N. Hansen, The cma evolution strategy: A tutorial, arXiv preprint arXiv:1604.00772 (2016).

[8] R. C. Barros, M. P. Basgalupp, A. C. De Carvalho, and A. A. Freitas, A survey of evolutionary algorithms for decision-tree induction, IEEE Transactions on Systems, Man, and Cybernetics, Part C (Applications and Reviews) **42**, 291 (2011).

[9] J. Bergstra and Y. Bengio, Random search for hyper-parameter optimization., Journal of machine learning research **13** (2012).

[10] R. W. Draft, M. R. McGill, V. Kapoor, and V. N. Murthy, Carpenter ants use diverse antennae sampling strategies to track odor trails, Journal of Experimental Biology **221**, 10.1242/jeb.185124 (2018).

[11] P. G. Hepper and D. L. Wells, How many footsteps do dogs need to determine the direction of an odour trail?, Chemical Senses **30**, 291 (2005).

[12] D. G. Wallace, B. Gorny, and I. Q. Whishaw, Rats can track odors, other rats, and themselves: implications for the study of spatial behavior, Behavioural brain research **131**, 185 (2002).

[13] V. Mnih, K. Kavukcuoglu, D. Silver, A. Graves, I. Antonoglou, D. Wierstra, and M. Riedmiller, Playing atari with deep reinforcement learning, arXiv preprint arXiv:1312.5602 (2013).

[14] A. Y. Ng, D. Harada, and S. Russell, Policy invariance under reward transformations: Theory and application to reward shaping, in *ICML*, Vol. 99 (1999) pp. 278–287.

[15] Y. Hu, W. Wang, H. Jia, Y. Wang, Y. Chen, J. Hao, F. Wu, and C. Fan, Learning to utilize shaping rewards: A new approach of reward shaping, Advances in Neural Information Processing Systems **33**, 15931 (2020).

[16] E. Wiewiora, Potential-based shaping and q-value initialization are equivalent, Journal of Artificial Intelligence Research **19**, 205 (2003).

[17] J. Schulman, F. Wolski, P. Dhariwal, A. Radford, and O. Klimov, Proximal policy optimization algorithms, arXiv preprint arXiv:1707.06347 (2017).

[18] A. Raffin, A. Hill, A. Gleave, A. Kanervisto, M. Ernestus, and N. Dormann, Stable-baselines3: Reliable reinforcement learning implementations, Journal of Machine Learning Research **22**, 1 (2021).

[19] G. Reddy, B. I. Shraiman, and M. Vergassola, Sector search strategies for odor trail tracking, Proceedings of the National Academy of Sciences **119**, e2107431118 (2022).

[20] M. Vergassola, E. Villermaux, and B. I. Shraiman, 'infotaxis' as a strategy for searching without gradients, Nature **445**, 406 (2007).

[21] T. Matiisen, A. Oliver, T. Cohen, and J. Schulman, Teacher-student curriculum learning, IEEE transactions on neural networks and learning systems **31**, 3732 (2019).

[22] V. Mnih, K. Kavukcuoglu, D. Silver, A. A. Rusu, J. Veness, M. G. Bellemare, A. Graves, M. Riedmiller, A. K. Fidjeland, G. Ostrovski, *et al.*, Human-level control through deep reinforcement learning, nature **518**, 529 (2015).
